# Intranasal Lidocaine Administration via Mucosal Atomization Device: A Simple and Successful Treatment for Postdural Puncture Headache in Obstetric Patients

**DOI:** 10.3390/biomedicines11123296

**Published:** 2023-12-13

**Authors:** Benedikt Hermann Siegler, Rui Pedro dos Santos Pereira, Jens Keßler, Stephanie Wallwiener, Markus Wallwiener, Jan Larmann, Susanne Picardi, Richard Carr, Markus Alexander Weigand, Beatrice Oehler

**Affiliations:** 1 Department of Anesthesiology, Medical Faculty Heidelberg, Heidelberg University, Im Neuenheimer Feld 420, D-69120 Heidelberg, Germany; ruipedro.dossantospereira@med.uni-heidelberg.de (R.P.d.S.P.); jens.kessler@med.uni-heidelberg.de (J.K.); jan.larmann@med.uni-heidelberg.de (J.L.); susanne.picardi@med.uni-heidelberg.de (S.P.); markus.weigand@med.uni-heidelberg.de (M.A.W.); beatrice.oehler@med.uni-heidelberg.de (B.O.); 2 Department of General Gynecology and Obstetrics with Polyclinic, Women’s Hospital, Medical Faculty Heidelberg, Heidelberg University, Im Neuenheimer Feld 440, D-69120 Heidelberg, Germany; geburtshilfe@uk-halle.de (S.W.); gyn@uk-halle.de (M.W.); 3 Department of Anesthesiology, Medical Faculty Heidelberg, Universitaetsmedizin Mannheim, Heidelberg University, Ludolf-Krehl-Str. 13-17, D-68167 Mannheim, Germany; richard.carr@medma.uni-heidelberg.de

**Keywords:** local anesthetics, topical treatment, midwifery, pain, epidural anesthesia, accidental dural puncture, sphenopalatine ganglion

## Abstract

(1) Background: Postdural puncture headache (PDPH) remains a serious complication in obstetric patients. While the epidural blood patch represents the current gold standard in therapy, a growing number of alternative measures are thought to be beneficial for clinical management. The purpose of this study was to retrospectively analyze the efficacy of intranasal lidocaine administration to treat PDPH in obstetrics at our university hospital; (2) Methods: A retrospective analysis of the medical records of patients with PDPH has been performed focusing on the techniques of administration, dosing, treatment duration, impact on pain intensity as well as side effects of intranasal lidocaine; (3) Results: During the study period, 5610 obstetric patients received neuraxial anesthesia, of whom 43 (0.77%) developed PDPH. About one third of the patients with PDPH after spinal anesthesia (*n* = 8), epidural anesthesia (*n* = 5) or both (*n* = 2) were treated with intranasal lidocaine. Lidocaine was administered either via gauze compresses (GC, *n* = 4), a mucosal atomization device (MAD, *n* = 8) or with a second-line mucosal atomization device due to low gauze compress efficacy (*n* = 3). All patients treated with lidocaine refused the epidural blood patch. Nebulization of lidocaine resulted in a significant reduction in pain intensity after the first dose (*p* = 0.008). No relevant side effects developed except sporadic temporal pharyngeal numbness. The utilization of the mucosal atomization device averted the necessity for an epidural blood patch, whether employed as the primary or secondary approach; (4) Conclusions: Our data imply that the mucosal atomization device enhances the efficacy of intranasal lidocaine administration in obstetric patients suffering from PDPH.

## 1. Introduction

In Western countries, spinal and epidural anesthesia (SpA and EDA) are used in up to 83% of obstetric patients for caesarian sections or pain relief during labor [1,2,3,4]. Postdural puncture headache (PDPH) represents a severe complication following neuraxial analgesia with an incidence of 0.5% to 0.7% [5,6] and reported rates of 50–80% after dural perforation upon EDA [7,8]. The relevance of PDPH especially for the obstetric population is underscored by the fact that risk is higher among women than men, regardless of the technique being used [9].

A recently published 10-year retrospective observational study analyzed adverse events following accidental dural puncture upon labor EDA. With 53.6%, PDPH represented the most frequent complication in the postpartum period [8].

Symptoms of PDPH typically become exacerbated in an upright body position and develop within three days after dural puncture [10], although more delayed onsets have also been described [11]. In more than one third of all cases, PDPH results in a significant loss of physical ability [12], which not only affects maternal care but may also decrease the acceptance of neuraxial techniques during later pregnancies [13]. Furthermore, accidental dural puncture and/or PDPH are associated with an increased risk of long-term morbidities, including chronic pain and depression [14].

Unfortunately, clinical management of PDPH remains a challenge [15]. Low effectiveness for conservative measures, including hydration, bed rest, analgesics, or caffeine has been reported [10]. If conservative treatments do not result in a timely relief of symptoms, current guidelines recommend the implementation of an autologous epidural blood patch (EBP) [16,17]. With success rates between 77 and 96% [18], the EBP represents the current gold standard in PDPH therapy. Rare, but severe side effects of this treatment include nerve damage, subdural hematoma, or spinal infections. In addition, the EBP procedure can be painful and PDPH symptoms may become even worse [19]. In a multicenter trial by Gupta et al., implementation of an EBP was associated with a fast reduction in pain intensity; however, in 20% of patients a second blood patch was needed [20].

Growing numbers of various medications and (non)invasive procedures have been discussed to be beneficial in the management of PDPH, including intravenous neostigmine and atropine [21], epidural morphine [22], acupuncture [23,24], greater occipital nerve block, or sphenopalatine ganglion block [25,26,27,28,29,30]. We demonstrated in a case report that the noninvasive nasal administration of lidocaine using a mucosal atomization device (MAD) provides immediate and persisting symptom relief in obstetric patients suffering from PDPH [31]. Intranasal lidocaine is generally well-received, deemed safe, and typically entails only minor side effects. In our previous case report, only numbness of the throat has been reported as unpleasant [31].

So far, the efficacy and safety of intranasal lidocaine via an MAD in parturient women with PDPH have not been tested in a systematic approach. The aim of this retrospective chart review was the assessment of the efficacy as well as potential side effects of intranasal lidocaine for the treatment of PDPH after obstetric neuraxial anesthesia at a single institution.

## 2. Materials and Methods

### 2.1. Ethical Approval of the Study Protocol

The retrospective study was performed in accordance with the Declaration of Helsinki after approval by the Ethics Committee of the Medical Faculty of Heidelberg University (Alte Glockengießerei 11/1, D-69115 Heidelberg, Germany; approval number: S-566/2020; date of approval: 18 August 2020). Study-related formal consent was not required.

### 2.2. Data Collection

The retrospective analysis was conducted to evaluate the efficacy of intranasal lidocaine administration to treat PDPH after obstetric neuraxial anesthesia at Heidelberg University Hospital. Data were extracted from electronic and paper-based records. The electronic hospital information system i.s.h.med^®^ (Cerner Health Services Deutschland GmbH, Berlin, Deutschland; 770 final release, version 7700.1.12.3395) as well as the documentation software Medlinq^®^ (Medlinq Softwaresysteme GmbH, Hamburg, Deutschland; version 20.4.1) for post-puncture visitations were used to identify obstetric patients presenting with PDPH.

### 2.3. Study Population, In- and Exclusion Criteria

All parturient women older than 18 years who received neuraxial analgesia (i.e., SpA or EDA) during labor or cesarean section between February 2017 and August 2021 were included in the retrospective data analysis. The patients provided formal consent for obstetric regional anesthesia after receiving a comprehensive explanation of the procedure, possible complications such as PDPH as well as potential treatment options. These included the epidural blood patch, the current gold standard in therapy, conservative treatment options like caffeine and non-opioid analgesics as well as the topical application of lidocaine.

Cases were screened for the occurrence of PDPH following neuraxial analgesia and were included in the final analysis when intranasal lidocaine administration was used to treat this complication. The diagnosis of PDPH was retrospectively confirmed based on medical records and according to current consensus guidelines [16,32]. Patients without PDPH diagnoses and those without intranasal lidocaine treatment were excluded from the final analysis.

### 2.4. Intranasal Application of Lidocaine

Lidocaine 2% was applied by bilateral instillation of gauze compresses soaked with lidocaine (100 mg) in the nasal cavity and/or by bilateral nebulization (up to 60 mg) using a mucosal atomization device (MAD Nasal^TM^, Teleflex Medical, Morrisville, NC, USA). For the treatment, patients were placed in a lying position; however, when possible, the upper part of the body was raised if tolerated by the patients.

### 2.5. Study-Related Data and Outcome Measures

Evaluated data included demographics, history of PDPH and related risk factors, type of regional anesthesia, as well as the diagnostic criteria, severity and clinical management of PDPH. Techniques for intranasal lidocaine administration, dosing, treatment duration and impact on pain intensity were investigated and the efficacy of various treatment options was compared. Pain intensity was evaluated using the recorded values of an 11-point numeric rating scale (NRS, from zero = “no pain” to 10 = “worst possible pain”).

Outcome measures of interest were the reduction of pain intensity after the first dose and the prevalence of pain improvement. NRS scores ≤ 3 within 1 h (defined as ‘rapid improvement’) and within 24 h following intranasal lidocaine administration were defined as an improvement. Additional outcome measures were the type and prevalence of adverse events following lidocaine administration, recurring symptoms during the hospital stay or the need for an EBP.

### 2.6. Statistical Analysis

Statistical analysis and data visualization were performed using GraphPad Prism (Version 9.1.2, GraphPad Software, La Jolla, CA, USA). For descriptive statistics, variables are presented as medians and ranges in the case of continuous data or as absolute and relative frequencies in the case of categorical data. The nonparametric Wilcoxon signed-rank test was used to compare NRS scores before and after treatment. *p* values < 0.05 were considered significant.

## 3. Results

### 3.1. Patients’ Characteristics and Clinical Management of PDPH

**Figure 1:**
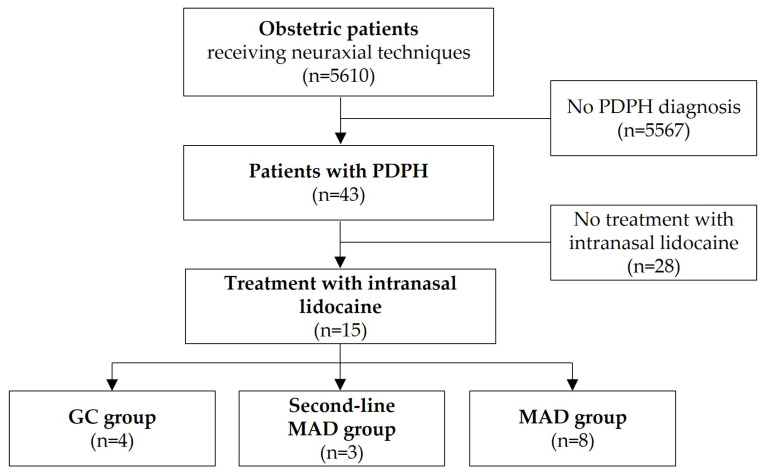
Study cohort. Abbreviations: GC gauze compresses, MAD mucosal atomization device, PDPH postdural puncture headache.

Throughout the study period, 5610 parturient women received neuraxial analgesia during labor or cesarean section, of whom 43 (0.77%) developed PDPH (Figure 1). Patients presenting with PDPH symptoms were visited at least once a day and carefully evaluated for other causes of headache. All patients immediately received conservative treatments such as oral/IV fluid intake, caffeine and analgesics like ibuprofen in line with our standard operating procedures. Thus, regarding the initial conservative treatment regime, no differences could be discovered in patients with PDPH. Drugs were given until symptoms had been improved during their hospital stay. An EBP was discussed at an early stage as a therapeutic option especially if symptoms persisted for more than 24 h after implementing conservative measures. Some patients were not willing to undergo an EBP or had contraindications to another puncture, hence alternative treatment options including intranasal lidocaine administration were offered. In total, we identified 15 patients treated with intranasal lidocaine (Figure 1).

PDPH occurred either after SpA (*n* = 8 patients) or EDA (*n* = 5 patients) with the majority of patients necessitating at least two puncture attempts. Two patients received SpA for secondary cesarean section after failed epidural attempts or after EDA with insufficient pain relief. Table 1 provides details on the demographics and clinical characteristics of the study cohort.

### 3.2. Intranasal Lidocaine Administration

Treatment consisted either of convoluted gauze compresses that were placed in both nasal cavities for 10–30 min after being soaked with lidocaine 2% (‘GC group’, *n* = 4) or bilateral nebulization of lidocaine 2% using a MAD device (‘MAD group’, *n* = 8). In three cases, MAD was used as second-line treatment due to the low efficacy of the GC therapy (‘Second-line MAD group’, Figure 1). The reported lidocaine dosages per nostril were 100 mg in patients receiving GC-treatment and 30–60 mg in patients receiving MAD-treatment, respectively. Table 2 summarizes the details on intranasal lidocaine administration and the outcomes of the study cohort.

### 3.3. Reduction of Pain Intensity

**Figure 2:**
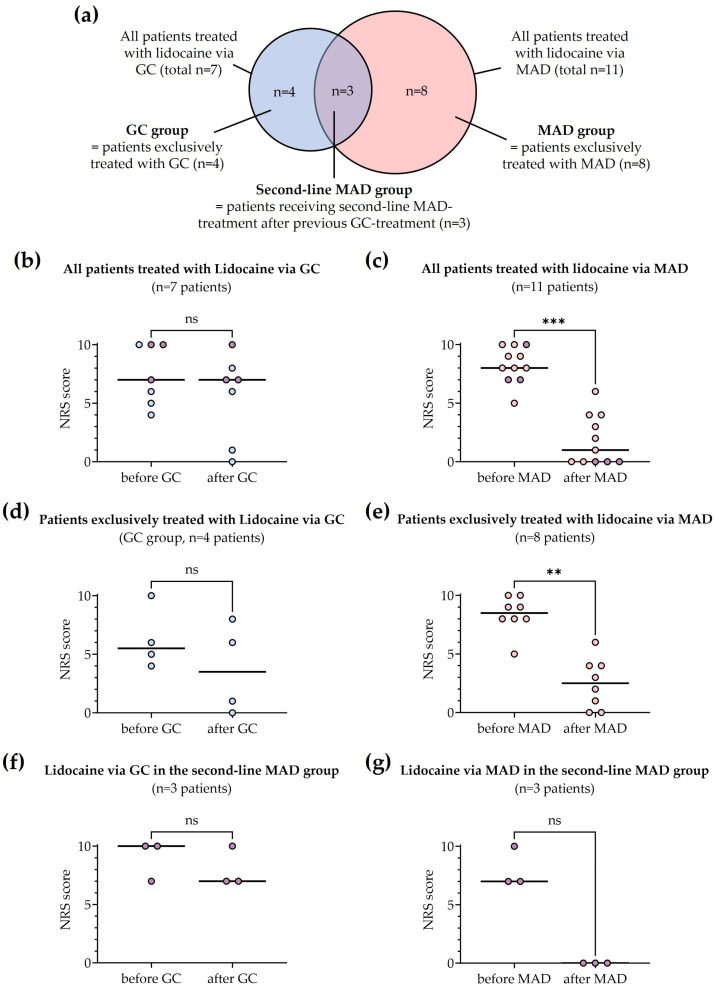
Reduction in pain scores (NRS) after first dose of intranasal lidocaine 2%. Colors indicate the different groups: Patients exclusively treated with lidocaine via GC (blue), patients exclusively treated with lidocaine via an MAD (light red) and patients first treated with lidocaine via GC, followed by administration via an MAD (purple). (**a**) Venn-diagram showing the distribution of treatments among patients. (**b**) All patients treated with lidocaine via GC (*n* = 7 patients; *p* = 0.13). (**c**) All patients treated with lidocaine via an MAD (*n* = 11 patients; *p* = 0.001). (**d**) Patients exclusively treated with lidocaine via GC (*n* = 4 patients; *p* = 0.25). (**e**) Patients exclusively treated with lidocaine via an MAD (*n* = 8 patients; *p* = 0.0078). (**f**) Patients receiving lidocaine via GC in the second-line MAD group (*n* = 3 patients; *p* > 0.99). (**g**) Patients receiving lidocaine via an MAD in the second-line MAD group (*n* = 3 patients; *p* = 0.25). Abbreviations: GC gauze compresses, MAD mucosal atomization device, NRS numeric rating scale. Horizontal lines represent the median of each group. Differences in group comparisons are marked with ‘**’ (*p* values ≤ 0.005), ‘***’ (*p* values ≤ 0.001) or ‘ns’ (not significant), respectively.

Nebulization of lidocaine via an MAD significantly reduced pain intensity after the first dose. Analysis of pain scores recorded before and after treatment (Figure 2) revealed a median of differences in the NRS score of −6 in all patients treated with MAD (MAD group and Second-line MAD group, *n* = 11 patients, *p* = 0.001, Figure 2a).

Regarding patients exclusively treated with the nebulization of lidocaine, we found the same median of differences in the NRS score of −6 (MAD group, *n* = 8 patients; *p* = 0.008, Figure 2b). In the three patients of the Second-line MAD group, a median of differences in the NRS score of −7 was observed (Figure 2c). No significant reduction of pain intensity was observed after the first dose of lidocaine via GC (median of differences −2, Figure 2d; median of differences −3, Figure 2e; median of differences 0.0, Figure 2f).

A reduction in pain intensities to NRS scores ≤ 3 within 24 h was achieved in 10/15 cases. These included 5 of 8 patients in the MAD group, 50% of patients in the GC group and all patients receiving nebulized lidocaine as a second-line MAD-treatment.

Rapid effects (NRS score ≤ 3 within 1 h) were observed in five patients and exclusively occurred after MAD therapy.

In eight patients (6 of 8 patients in the MAD group and 2 of 4 patients in the GC group) a single dose of intranasal lidocaine resulted in persisting pain relief. Persisting pain relief was also observed in the three patients receiving lidocaine via an MAD as second-line treatment.

### 3.4. Adverse Events, Recurring Symptoms and Need for an EBP

Except for sporadic reports of temporal pharyngeal numbness due to swallowed lidocaine, no other relevant side effects were observed. None of the patients displayed persisting or recurring symptoms or required an EBP.

## 4. Discussion

Postpartum headache due to neuraxial anesthesia not only impairs the mother’s general well-being, but also affects her care for the newborn child. Although PDPH is often self-limiting, long-term sequelae including head-, neck- and backache as well as depression have been reported in the obstetric population [14,33]. These results underscore the urgent need for the fast and efficient treatment of PDPH.

For the first time, we investigated the practicality of an MAD as a beneficial device to successfully apply lidocaine intranasally for the treatment of PDPH in a systematic approach. The current gold standard, the EBP, is highly effective [20]. We offer this treatment to our patients if conservative treatments do not result in a timely relief of symptoms. Nonetheless, there is an urgent need for effective alternative treatment options, especially in case of rejection or other contraindications that hinder EBP implementation. In our experience, the invasiveness of the EBP commonly lowers its acceptance among many patients, especially because of the same procedure being needed as the one causing the headache. Moreover, an EBP can be painful and is again associated with possible severe complications. Hence, we regularly discuss potential alternatives with our patients, including intranasal lidocaine administration. In this study, we describe the utility of intranasal lidocaine to treat PDPH in obstetric patients at our hospital. All included patients refused an EBP and were willing to undergo alternative treatment approaches including intranasal lidocaine administration. Our data indicate that lidocaine nebulization via an MAD is a simple and effective treatment option and represents a beneficial alternative to the invasive EBP, that results in a significant reduction of pain intensity and prevents the need for invasive measures, including an EBP.

According to the current state of knowledge, two major mechanisms seem to underlie PDPH pathophysiology: on the one hand, the loss of cerebrospinal fluid (CSF) causes the irritation of meninges, nerves, and vessels resulting in the characteristic position-dependent pain. On the other hand, reduced CSF volume induces compensatory intracranial vasodilation that, in turn, further promotes headache [10]. The sphenopalatine ganglion (SPG), a junction of sensory and autonomic fibers located posterior to the maxillary sinus, seems to play a central role in various forms of pain, including PDPH [34]. Due to its localization, the ganglion is a well-known target for topical treatments. In the case of PDPH, an SPG blockade is assumed to reduce parasympathetic impulses, thereby counteracting the pain resulting from intracranial vasodilation. Moreover, a modulatory effect on neurogenic inflammatory mediators has been postulated [35].

A recent meta-analysis of randomized controlled trials (including non-obstetric patients) revealed the SPG blockade to be superior to conservative measures concerning short-term pain improvement [36]. Interestingly, carrying out the block within the first 24 h after PDPH diagnosis was found to reduce the risk of symptom recurrence as well as the time to discharge compared to a later treatment. However, early versus late timing did not impact pain improvement [37].

Several trials have analyzed the value of the SPG blockade for the treatment of PDPH in the obstetric population [25,28,29,30]. While several techniques have been described to perform the block [38], most studies use cotton tips placed in the nasal cavity near the pterygopalatine fossa. However, the exact positioning of the tips may be impeded, for instance, in cases of nasal polyps or septum deviation. Moreover, the procedure can cause the irritation and bleeding of the nasal mucosa and may thus be contraindicated in some patients. To avoid any discomfort or adverse effects, we modified the technique using soft GC that were soaked with lidocaine and carefully placed in both nasal cavities for 10–30 min. In three cases, pain intensity was not sufficiently reduced using GC, so we offered further treatment options including the use of an MAD and escalation via an EBP. All three patients decided for the alternative approach via second-line MAD therapy and experienced complete and persisting pain relief. Based on these results, we subsequently favored the MAD for intranasal lidocaine treatments instead of the GC in our patients. As an important finding of this study, in contrast to application via GC, nebulization of 2% lidocaine significantly reduced the pain intensity already after the first dose. In line, rapid treatment effects (<1 h) were exclusively observed after MAD therapy.

Intranasal lidocaine is generally well-received, deemed safe, and typically entails only minor side effects, including throat numbness, a stinging sensation, and a bitter taste. Cases where cardiovascular or neurotoxic adverse events after topical application of lidocaine are unavailable. In our study, only the numbness of the throat has been reported as unpleasant.

Mechanisms underlying the efficacy of intranasal lidocaine, especially after administration via an MAD as in this study, remain unclear. Due to variations in application techniques and the nasal cavity’s cavernous nature, determining tissue concentrations presents challenges. Lidocaine concentrations applied to the nose show a tenfold variability across studies, with reported volume variations reaching 100 times. For instance, pump sprays typically dispense approximately 80 to 100 µL per puff. In contrast, the recommended total volume for TX360^®^ application is 600 µL, while the volumes used with SpheoCath^®^ or MAD are at least ten times greater than a single puff, exceeding >1000 µL [31,39,40]. In studies involving healthy male volunteers, the absorption and bioavailability of 100 mg intranasally applied lidocaine gel exhibited a tenfold variation, with a maximum of 50% plasma uptake within an hour of application [41]. Additionally, placebo effects can be substantial, complicating the determination of indices based on effective concentration [30].

Studies comparing the effectiveness of various lidocaine concentrations have not demonstrated dose-dependent kinetics, suggesting that the concentrations used may be supramaximal. Interestingly, there appears to be a potential correlation between efficacy and the volumes administered. Higher volumes of lidocaine may lead to more effective treatment. In rat studies, intranasal lidocaine achieved higher levels in brain tissue and cerebrospinal fluid compared to intravenous injection [42].

In our study, lidocaine concentrations were neither determined in plasma nor in CSF samples. Potential target sites remain speculative. Nevertheless, several important findings might support certain modes of action: First, we found differences in pain reduction comparing patients receiving lidocaine via an MAD with those treated with soaked gauze compresses. Since the latter were left up to 30 min in both nasal cavities, it can be assumed that a certain proportion of lidocaine (potentially exceeding the dose of the MAD-therapy) was absorbed by the nasal mucosa. Nevertheless, lidocaine nebulization showed stronger effects on pain reduction. Second, rapid pain improvement exclusively occurred after MAD therapy with two patients reporting NRS scores ≤ 3 after 5 and 10 min, respectively. Third, in three cases (including the two patients with immediate pain relief), we observed a beneficial effect of the MAD therapy, although these patients did not respond to the previous treatment via GC. A possible explanation could be that the nebulized lidocaine reached areas within the nasal cavity that were not or only insufficiently covered by the inserted compresses.

Numerous case reports suggest positive outcomes with intranasal lidocaine in headache patients. While a significant percentage of patients respond well, there is also a substantial proportion of non-responders [43]. Although topical lidocaine to the sphenopalatine ganglion has been considered promising for treating acute cluster headache attacks, only one-third of patients seem to benefit. It is unlikely that lidocaine exclusively acts on the sphenopalatine ganglion [44]. Possible additional structures may include the Vidian nerve, the maxillary branch of the trigeminal nerve, and olfactory sensory nerve terminals, which could be vital for preventing migraines by inhibiting the sense of smell—one of the potential auras. Nevertheless, lidocaine reabsorption from the nasal cavity into either the CSF or the bloodstream may also contribute to the observed reduction of symptoms. Among PDPH patients, approximately half of the published studies indicate that pain relief with intranasal lidocaine is not significantly different from placebo. Beyond headache disorders, intranasal lidocaine has been reported to be beneficial in various pain conditions, including facial (trigeminal) neuralgia, temporomandibular joint dysfunction, and tinnitus. It has also shown promise in addressing more distant pain syndromes such as shoulder and neck pain, lower back pain, and complex regional pain syndrome [45,46,47,48,49,50,51]. Additionally, the systemic administration of lidocaine was shown to exert antinociceptive effects in acute and chronic pain states [52].

While evidence concerning the clinical effectiveness of lidocaine grows, the exact molecular mechanisms responsible for its antinociceptive effects are still under debate. Besides a blockade of voltage-gated ion channels as the approved mode of action, several other targets of lidocaine involved in nociception have been identified, including N-methyl-D-aspartate and muscarinic cholinergic receptors [52,53]. Whether these mechanisms play a role in the long-term analgesic effects of lidocaine that were observed in some of our patients as well as in other (pre-) clinical studies [54,55] needs to be elucidated. Interestingly, the modulation of some of these targets necessitates far lower lidocaine concentrations than those required for a sodium channel blockade [52].

To date, only little is known about the bioavailability of intranasal lidocaine in humans. One study including six healthy individuals found highly variable plasma concentrations of lidocaine after intranasal administration using a gel preparation [41]. Murine studies indicate different nasal absorption depending on the formulations used [56], though, future clinical studies should address the pharmacokinetics of lidocaine, including its bioavailability and elimination after nasal administration via an MAD.

In addition to effectively and rapidly reducing pain, giving lidocaine via an MAD offers several advantages for treating PDPH, not only in the obstetric population. First, the simple and completely noninvasive technique can be used immediately at the patients’ bedside. Second, compared to the EBP, MAD therapy requires far fewer resources. Third, toxic concentrations due to systemic resorption of lidocaine can be avoided by using MAD systems, which allow individual dosing. Notably, except for reports of temporal pharyngeal numbness due to swallowed lidocaine, no relevant side effects developed in our patients.

We acknowledge several limitations of this study. The analysis of medical records was conducted retrospectively. Beside the immanent limitations of such a study design, it enabled us to investigate a relatively high number of 5610 parturient women receiving neuraxial analgesia during labor or cesarean section with comparable periinterventional regimes. The PDPH rate of 0.77% in our collective (*n* = 43 patients) explains the putative small number of investigated patients receiving intranasal lidocaine treatment. Moreover, both electronic and paper-based medical records were reviewed to confirm PDPH diagnosis. Therefore, automated data extraction was not feasible. However, using specific documentation software for post-puncture visitations as a data source, in our view, reduces the risk of information bias. Next, while it is unlikely that we missed a large proportion of patients suffering from PDPH during their hospital stay, we cannot rule out to have missed some patients with PDPH occurring after hospital discharge. Finally, since we exclusively investigated parturient women, the findings of our study are not generalizable to non-obstetric patients, i.e., those presenting with PDPH after diagnostic lumbar punctures or neurosurgical procedures. Despite these limitations, the results of our study imply that intranasal lidocaine administration might serve as a potential therapeutic alternative to treat PDPH. Experimental as well as prospective multicenter clinical studies are needed to validate our findings, to confirm the safety of this method and to shed light into the so far unknown physiology behind it.

## 5. Conclusions

Taken together, intranasal lidocaine nebulization resulted in a significant reduction of pain intensity in obstetric patients suffering from PDPH and prevented the need for invasive measures including an EBP. Therefore, this method might represent a simple treatment alternative, especially when an EBP is rejected by the patients or otherwise contraindicated. Although our findings need to be prospectively validated, we acknowledge the challenge in designing an appropriate study for this purpose in the obstetric population. Due to the retrospective design of this study, further research is necessary to gain insights about the mechanisms underlying the observed effects.

## Figures and Tables

**Table 1:** Demographics and clinical characteristics, presented as medians (ranges) or numbers (percents). Abbreviations: GC gauze compresses, MAD mucosal atomization device, NRS numeric rating scale, PDPH postdural puncture headache.

Variables	Total (*n* = 15 Patients)	GC Group (*n* = 4 Patients)	Second-Line MAD Group (*n* = 3 Patients)	MAD Group (*n* = 8 Patients)
Age (years)	32 (24 to 40)	30 (26 to 40)	34 (24 to 36)	33 (29 to 40)
Body weight (kg)	71 (57 to 179)	80 (58 to 179)	80 (60 to 140)	71 (57 to 95)
Height (cm)	163 (158 to 182)	161 (158 to 170)	160 (160 to 160)	170 (150 to 182)
Body mass index (kg/m^2^)	27 (21 to 70)	29 (22 to 70)	31 (23 to 55)	25 (21 to 32)
Risk factors for PDPH				
Age 20–30 years	6 (40%)	3 (75%)	1 (33%)	2 (25%)
Body mass index < 20 kg/m^2^	0 (0.0%)	0 (0.0%)	0 (0.0%)	0 (0.0%)
History of headache/migraine	3 (20%)	1 (25%)	1 (33%)	1 (13%)
History of PDPH	3 (20%)	1 (25%)	1 (33%)	1 (13%)
Neuraxial technique				
Spinal anesthesia	8 (53%)	2 (50%)	2 (67%)	4 (50%)
Epidural anesthesia	5 (33%)	0 (0.0%)	1 (33%)	4 (50%)
Both ^a^	2 (13%)	2 (50%)	0 (0.0%)	0 (0.0%)
Time from puncture to PDPH onset (days)	1.0 (0.0 to 3.0)	1.5 (1.0 to 3.0)	1.0 (1.0 to 1.0)	1.5 (0.0 to 3.0)
Pain intensity at time of PDPH diagnosis (NRS score)	7 (2 to 10)	7 (5 to 10)	6 (3 to 10)	8.5 (2 to 10)
PDPH-associated symptoms				
Neck stiffness/pain	12 (80%)	4 (100%)	2 (67%)	6 (75%)
Tinnitus/hearing loss	3 (20%)	0 (0.0%)	0 (0.0%)	3 (38%)
Photophobia	1 (6.7%)	0 (0.0%)	0 (0.0%)	1 (13%)
Nausea/vomiting	4 (27%)	2 (50%)	0 (0.0%)	2 (25%)
Conservative treatment				
Oral/IV fluid intake	9 (60%)	3 (75%)	1 (33%)	5 (63%)
Caffeine	12 (80%)	3 (75%)	3 (100%)	6 (75%)
Ibuprofen	12 (80%)	3 (75%)	3 (100%)	6 (75%)
Paracetamol	8 (53%)	2 (50%)	1 (33%)	5 (63%)

^a^ Two patients received spinal anesthesia for secondary cesarean section after failed epidural attempts or after epidural anesthesia with insufficient pain relief.

**Table 2:** Neuraxial technique, PDPH treatment, and outcomes. Abbreviations: ADP accidental dural puncture (noticed during puncture), BMI body mass index, EBP epidural blood patch, EDA epidural anesthesia, GC gauze compresses, MAD mucosal atomization device, PDPH postdural puncture headache, SpA spinal anesthesia.

Patient	Technique (Needle Size)	Attempts/ Risks	Day of PDPH Diagnosis ^a^	Imaging/Neurol. Review	EBP	Day of Intranasal Lidocaine Started ^b^	Treatment Group	Number of Treatments	NRS ≤ 3 within 1 h	NRS ≤ 3 within 24 h
1	EDA (18G) SpA (25G)	Multiple ^c^	2	No	refused	4	GC	1	No	Yes
2	EDA (18G) SpA (25G)	Multiple ^d^	1	No	refused	4	GC	3 per day, 3 days	No	No
3	SpA (25G)	4 attempts 2 levels	1	No	refused	2	GC	1	No	No
4	SpA (25G)	1 attempt, BMI 69.9	3	No	refused	3	GC	1	No	Yes
5	SpA (25G)	1 attempt	1	No	refused	1	Second-line MAD	1xGC, 1xMAD (next day)	No	Yes (after MAD)
6	EDA (18G)	4 attempts, 2 levels	1	No	refused	2	Second-line MAD	1xGC, 1xMAD (next day)	Yes (after MAD)	Yes (after MAD)
7	SpA (22G)	2 attempts, 2 levels, BMI 54.7	1	No	refused, too difficult	1	Second-line MAD	1xGC 1xMAD (same day)	Yes (after MAD)	Yes (after MAD)
8	SpA (25G)	2 attempts, 1 level	3	No	refused	3	MAD	1	Yes	Yes
9	SpA (25G)	1 attempt	1	No	refused	1	MAD	1	Yes	Yes
10	SpA (25G)	2 attempts, 1 level	1	No	refused	4	MAD	1	No	Yes
11	EDA (18G)	3 attempts, 1 level	2	No	refused	3	MAD	1	No	No
12	EDA (18G)	2 attempts, 1 level	3	No	refused	4	MAD	1	No	Yes
13	EDA (18G)	2 attempts, 2 levels, ADP	0	No	refused	1	MAD	3 per day, 3 days	No	No
14	SpA (25G)	2 attempts, 1 level	1	No	refused	1	MAD	1	Yes	Yes
15	EDA (18G)	2 attempts 2 levels, ADP	2	No	refused	2	MAD	2 within 3 h	No	No

^a^ Time from puncture to diagnosis; ^b^ time from puncture to treatment with intranasal lidocaine; ^c^ EDA not possible, no catheter placed; ^d^ EDA without relevant pain relief, secondary cesarean section under spinal anesthesia after removing EDA catheter.

## Data Availability

The data presented in this study are available on reasonable request from the corresponding author.
